# Hunting at the highway: traffic noise reduces foraging efficiency in acoustic predators

**DOI:** 10.1098/rspb.2010.2262

**Published:** 2010-11-17

**Authors:** Björn M. Siemers, Andrea Schaub

**Affiliations:** 1 Max Planck Institute for Ornithology, Sensory Ecology Group, Eberhard-Gwinner-Strasse, 82319 Seewiesen, Germany; 2 Animal Physiology, Institute for Neurobiology, University of Tübingen, Auf der Morgenstelle 28, 72076 Tübingen, Germany

**Keywords:** anthropogenic noise, sensory ecology, foraging, bats, masking, attention

## Abstract

Noise pollution from human traffic networks and industrial activity impacts vast areas of our planet. While anthropogenic noise effects on animal communication are well documented, we have very limited understanding of noise impact on more complex ecosystem processes, such as predator–prey interactions, albeit urgently needed to devise mitigation measures. Here, we show that traffic noise decreases the foraging efficiency of an acoustic predator, the greater mouse-eared bat (*Myotis myotis*). These bats feed on large, ground-running arthropods that they find by listening to their faint rustling sounds. We measured the bats' foraging performance on a continuous scale of acoustically simulated highway distances in a behavioural experiment, designed to rule out confounding factors such as general noise avoidance. Successful foraging bouts decreased and search time drastically increased with proximity to the highway. At 7.5 m to the road, search time was increased by a factor of five. From this increase, we predict a 25-fold decrease in surveyed ground area and thus in foraging efficiency for a wild bat. As most of the bats' prey are predators themselves, the noise impact on the bats' foraging performance will have complex effects on the food web and ultimately on the ecosystem stability. Similar scenarios apply to other ecologically important and highly protected acoustic predators, e.g. owls. Our study provides the empirical basis for quantitative predictions of anthropogenic noise impacts on ecosystem processes. It highlights that an understanding of the effects of noise emissions and other forms of ‘sensory pollution’ are crucially important for the assessment of environmental impact of human activities.

## 1. Introduction

Noise pollution from human traffic networks and industrial activity occurs in vast areas of our planet [1] and potentially affects wildlife over both terrestrial and aquatic environments [2,3]. A considerable body of research documents how anthropogenic noise impacts animal communication [4–6]. Some birds adjust pitch [7,8], amplitude [9] or timing [10] of their song to counteract masking, right whales change the tune of their communication calls in response to shipping noise [11] and male frogs lose acoustic space for attracting females to traffic noise [12].

**Figure 1.**
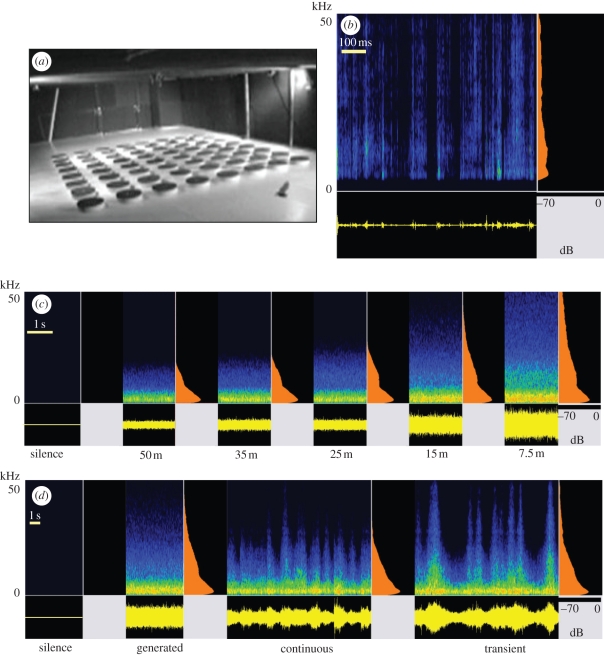
Experimental set-up and sound stimuli. (*a*) Video screenshot of the experimental foraging area. (*b*) Example of a carabid beetle walking sound that we used to signal prey to the bats. (*c*) Noise treatments in experiment 1; ‘silence’ as a control and digitally generated, standardized traffic noise corresponding to different distances to a highway (from the right, i.e. outer, lane. (*d*) Examples of the noise treatments in experiment 2; the digitally generated 15 m stimulus from experiment 1, recorded traffic noise as 15 m from a highway, but with silent intervals between passing cars cut out, unchanged recorded traffic noise as 15 m from a highway, and again ‘silence’. All sound examples in spectrogram representation with oscillogram below and averaged power spectrum on the right. Amplitude is colour coded (relative dB scale).

A more comprehensive understanding of how anthropogenic noise influences ecosystem processes, albeit urgently needed to devise mitigation measures [2,6], is only starting to emerge, however. Here, a crucially important question is how noise pollution affects predator–prey interactions, as these stand at the heart of ecosystem stability and dynamics. Recent evidence suggests that songbirds experience decreased predation rate in noisy environments [13], and hermit crabs are distracted by boat motor noise and hence less vigilant against approaching predators [14]. No study has as yet directly assessed how anthropogenic noise interacts with the foraging efficiency of a predator. We hypothesize that acoustic predators, such as owls [15], some carnivores and nocturnal primates [16], and many species of bat [17–19], that detect and localize animal prey by eavesdropping on their communication or locomotion sounds, are likely to experience reduced foraging success in noise, because it masks the prey cues. In the present study, we assessed for the first time, to our knowledge, anthropogenic noise impact on prey detection performance of an acoustic predator. We chose the greater mouse-eared bat (*Myotis myotis*) as a model species. These bats feed on large, ground-running arthropods such as carabid beetles, hunting spiders and centipedes [20] that they detect and track down by listening to the faint rustling sounds that the arthropods produce when walking [21,22]. Most of these arthropods are predators themselves and thus noise impact on the bats' foraging performance might have complex effects on the food web (B. M. Siemers, S. Greif, I. Borissov, S. L. Voigt-Heucke & C. C. Voigt 2010, unpublished data). Greater mouse-eared bats occur in most of Central and Southern Europe and can cover nightly foraging distances of more than 25 km [23]. Most of Europe's existing and planned highways thus cross potential mouse-eared bat foraging habitat. As the species is protected under the highest conservation category of the European Habitats Directive, the potential impact of traffic noise on the bats' foraging efficiency is of strong practical relevance. In laboratory experiments, these bats avoid loud, broadband noise, including playback of traffic noise corresponding to 10–15 m from a highway [24], but the reason for noise avoidance has not been studied. Here, we tested the hypothesis that traffic noise affects foraging efficiency in these bats, as a model for acoustic predators. In a large flight room, we set up an experimental foraging area with 64 platforms (figure 1*a*) in each of which we hid a loudspeaker that could play rustling sound of the bats' main prey—carabid beetles [20,22]—at naturalistic amplitudes [25] (figure 1*b*). The set-up mimicked the natural foraging scenario of these bats. As soon as they heard the prey walking sounds they landed briefly on the respective platform and picked up a food reward from above the speaker (see electronic supplementary material, video S1). We then applied different noise treatments through an array of broadband loudspeakers mounted on two sides of the experimental foraging area and conducted a total of 5069 1 min foraging trials with eight bats. It was not possible for the bats to avoid the noise, as the entire foraging area was ensonified. Thus, we could measure the bats' prey detection and localization performance under the noise profiles of a series of highway distances.

## 2. Material and methods

### (a) Animals and housing

Eight adult male greater mouse-eared bats (*Myotis myotis*) were used for experimentation. The animals were captured for these experiments under licence from Regierungspräsidium Freiburg (licence no. 55-8852.44/1095) and held and tested in specially designed facilities at the University of Tübingen (approved by Regierungspräsidium Tübingen). They were housed in a flight cage of 2 × 1.5 × 2 m (l × w × h) with an inverted light regime (8 h D: 16 h L) and tested during their activity phase. The bats had been on an inverted photoperiod for more than six months before the experiments started and thus were fully accustomed to it. All training and testing was conducted during the bats' natural activity period; i.e. during the (artificial) night. The bats received water ad libitum and food (mealworms, larvae of *Tenebrio molitor*) during the experiments, which were run 5 days a week. Their diet was also supplemented with crickets (*Schistocerca gregaria*) at the weekends, and with vitamins and minerals once every four weeks.

### (b) Experimental set-up

Bats were tested in a large flight room with dimensions of 13 × 6 × 2 m; walls and ceiling were covered with sound absorbing foam to reduce echoes and reverberations. In the middle of the room, 64 cylindrical platforms (diameter: 40 cm, height: 10 cm) were regularly arranged in a 4.6 × 4.6 m square (figure 1*a*). A plastic Petri dish was inserted on the centre of each platform. Below a hole in each dish, we hid a small, broadband speaker (Sennheiser HD 555/595) connected to a laptop via an external soundboard (RME Fireface 800 Interface, sampling rate 192 kHz). In each trial, we played a prey rustling sound from one of the 64 platforms. If the bat landed on the correct platform within 1 min from the onset of playback, it was allowed to take a mealworm from the dish. Mealworms were freshly killed by cooling and thus did not crawl or produce noise.

For the noise treatments, six broadband tweeter loudspeakers (Swans, RT2H_A, operational from 1–70 kHz; noise high-pass filtered at 1 kHz, see below) were mounted around the experimental foraging area; three on each of the two shorter sides of the rectangular flight room. They were driven using the RME Fireface 800 (sampling rate 192 kHz) and broadband amplifiers (WPA-600 Pro, Conrad Electronics). Files were played continuously throughout a trial.

Each bat was tested in each test condition 64 times, with the prey stimulus played from each of the platforms exactly once. This approach was chosen to factor out any interaction of the exact noise sound field and the prey location. We also avoided repeating any noise stimulus type more than three times in a row. Within these constraints, the sequence of stimuli and prey positions was randomized for each bat. With the exception of one bat that flew in only 13 of 64 trials for the 7.5 m treatment, all eight bats performed in all 64 trials of the six test conditions of experiment 1 and the four conditions of experiment 2, so that our results are based on a total of 5069 trials. We first performed experiment 1 and then experiment 2. Each bat was tested as long as it showed clear foraging motivation (resting bouts between trials less than 2 min).

Experiments were run in the dark with one bat at a time and filmed (Sanyo BW CCD camera VCB-3572 IRP, Computar lens M0518, Sony recorder GVD1000E) under infrared (IR) illumination (custom-made IR-strobes) for online display and video-taped for later off-line analysis.

### (c) Acoustic stimuli

All playback files were arranged or generated in Adobe Audition 1.5 (adobe) and had a sampling rate of 192 kHz, i.e. contained frequencies up to 96 kHz. All files were highpass-filtered at 1 kHz (digital fast Fourier transform filter, 2048 points, Blackman window) to remove sound probably not audible to the bats and to avoid damage to the speakers.

For experiment 1, an empty wav-file (amplitude values of all samples at zero) was generated for the ‘silence’ treatment. For the traffic noise treatments, we digitally generated noise that would correspond to the average loudest 0.5 s of a passing vehicle as experienced at 7.5, 15, 25, 35 and 50 m from the right (outer) lane of a highway. This approach was taken to have a standardized and representative traffic noise background. The average power spectral density of a passing vehicle was computed based on broadband recordings of 50 passing cars and 50 passing trucks at speeds of approximately 80 km h^−1^ at the Autobahn A8 close to Stuttgart, Germany, at 7.5 m distance (see [24] for details). For the four treatments that corresponded to larger distances, we calculated the decay of frequency and amplitude over distance and verified our calculations with empirical recordings [24]. High frequencies, which were already faint, decayed quickly with distance (comp. figure 1*c*). The playback files were filtered to compensate for the speaker characteristics and amplified so that the sound field at the experimental foraging area corresponded to the desired highway distances [24]. It is important to note that our treatments in experiment 1 mimicked a continuous stream of vehicles, as we played sound levels corresponding to the loudest 0.5 s of a passing vehicle for the entire 1 min trial.

For experiment 2, a representative 1 min recording of traffic noise at a highway was used (Autobahn A8; 29 passing vehicles per minute); for details see [24]. It was filtered to compensate for the speaker characteristics and amplified so that intensities at the experimental foraging area corresponded to 15 m next to the highway [24]. For the ‘transient’ treatment, it was left unchanged otherwise, i.e. the noise rose and fell as cars and trucks passed by. For the ‘continuous’ treatment, more silent parts were cut out so that the playback file consisted of a series of 1.5 s peak levels around the moment when vehicles passed the microphone. Silence treatment and 15 m treatment as in experiment 1.

As prey sound at the feeding platforms, we played back rustling sounds at naturalistic amplitudes [25] of typical mouse-eared bat prey. For this purpose, we had recorded four different individual ground beetles (*Carabus monilis*; 23–26 mm body lengths and 0.5–0.7 g) walking on moist leaf litter, a typical substrate in mouse-eared bat foraging areas, with a broadband, especially sensitive microphone (Type 40HH, G.R.A.S., Holte, Denmark); for details, see [25].

### (d) Data analysis

From the videos, we extracted whether a trial was successful, i.e. the bat landed on the correct platform, and if so, how long it took from onset of playback to landing (‘search time’). For each animal and test condition, we broke down all trials (generally 64) into a single value for each of the two behavioural variables to avoid pseudo-replication as follows.

— The proportion of successful trials, as displayed in the graphs. For statistical analysis, we transformed this proportion as follows in order to approximate a normal distribution [26],
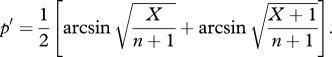
See [26, eqn. (13.8)], where *X* is the number of successful trials and *n* the total number of trials.
— Average search times over all trials per test condition.

Statistical tests were computed in SPSS 15.0.

## 3. Results

### (a) Experiment 1: effect of highway distance

**Figure 2.**
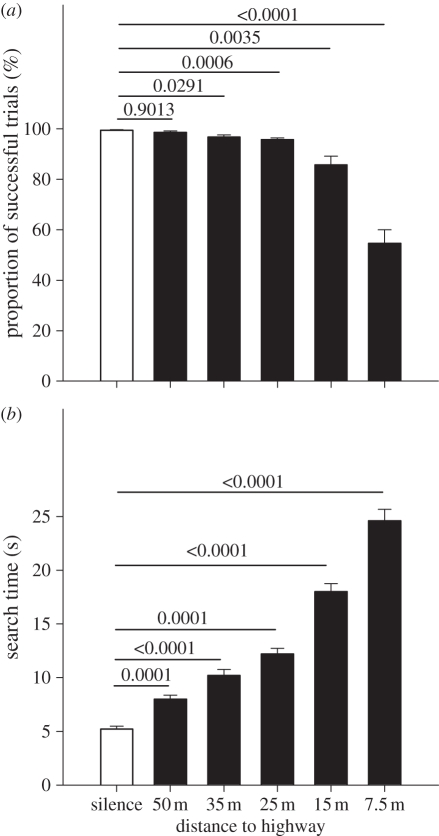
Foraging performance of the bats under noise conditions of different highway distances (experiment 1). (*a*) Proportion of successful foraging trials (prey found within a 1 min time window). (*b*) Search time; i.e. time from onset of prey rustling playback to the moment when the bat landed on the platform (only successful trials included). Means plus one standard error. *p*-values from paired *t*-test performed post hoc to a repeated measures ANOVA (see text) are indicated above the bars. We compared each highway distance to the silence treatment (control; white bar); *p*-values were Bonferroni corrected to account for the five pairwise comparisons. For noise stimuli, see figure 1*c*.

In experiment 1, we used computer-generated noise spectra that represented average traffic noise at different distances to a highway (figure 1*c*). The main energy of traffic noise is clearly within the human hearing range, largely below 5 kHz [12]. Yet, traffic noise does have an ultrasonic component that decays rapidly over distance [24]. Close to a highway, it strongly overlaps the frequency spectrum of prey rustling sounds (main energy 3–30 kHz [25]; (compare panels *b* and *c* in figure 1) and hence there is a strong potential for acoustic masking. Indeed, the bats showed a markedly decreased foraging performance under noise conditions as found close to a highway. First, the noise treatment had a significant effect on the proportion of successful foraging trials (figure 2*a*; repeated measures ANOVA, *F*_5,35_ = 85.71, *p* < 0.0001). While success rate was close to 100 per cent under the control condition (‘silence’), it was reduced to 54.6 per cent for 7.5 m from the highway (for post hoc tests see figure 2). This performance is still high above the 1.6 per cent chance level that results from our 64 potential prey locations. The noise treatment effect on our second behavioural parameter, search time, was even more profound (figure 2*b*; *F*_5,35_ = 157.47, *p* < 0.0001). Average search time in the control condition was 5.2 s, while it rose to 24.6 s for 7.5 m from the highway. Even at 50 m, search time was still significantly higher and at 150 per cent of search time under the control condition (figure 2*b*). Extrapolation of our results suggests traffic noise effects on the bats' prey detection ability up to about 60 m from the highway.

### (b) Experiment 2: a control for noise type and continuity

**Figure 3.**
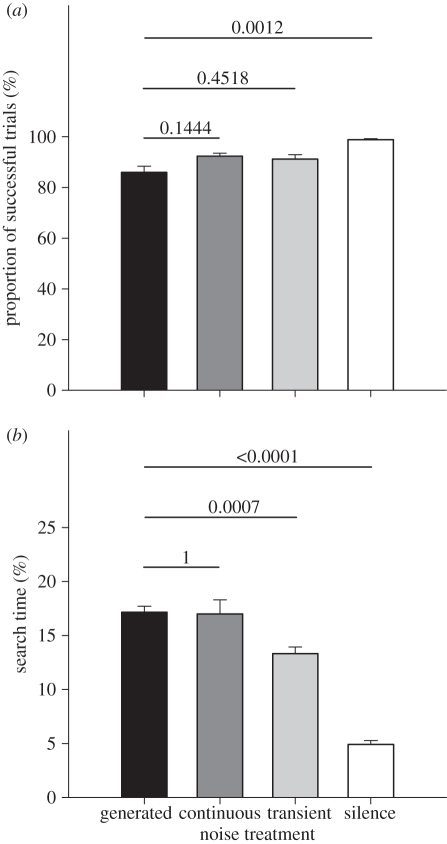
Foraging performance of the bats under digitally generated and recorded highway noise (experiment 2). (*a*) Proportion of successful foraging trials. (*b*) Search time. Means plus one standard error. *p*-values from paired *t*-tests performed post hoc to a repeated measures ANOVA (see text) are indicated above the bars. We compared each digitally generated playback stimulus with the 15 m treatment (‘generated’, black bar) as used in experiment 1; *p*-values were Bonferroni corrected to account for the three pairwise comparisons. For noise stimuli, see figure 1*d*.

We used the same 64 platform experimental foraging area and type of prey sound, but this time employed a different set of noise treatments (figure 1*d*). The aim was to compare the effect of the digitally generated noise stimuli from experiment 1—tailored to represent average highway noise [24]—with the effect of samples of real, recorded traffic noise. We thus repeated the 15 m treatment from experiment 1 and also played back two versions of traffic noise as recorded 15 m from a highway. One version was left unchanged (transient traffic noise), while in the other we cut out the silent intervals between passing cars (continuous traffic noise). The bats' performance in the 15 m treatment and in the silence treatment, which we also repeated, did not differ between experiments 1 and 2 (paired *t*-tests; 15 m, proportion of successful trials, *t*_7_ = 0.08, *p* = 0.9406; search time, *t*_7_ = 0.89, *p* = 0.4044; silence, proportion of successful trials, *t*_7_ = 2, *p* = 0.0856; search time, *t*_7_ = 1.02, *p* = 0.3430), which we take as evidence for the robustness and repeatability of our behavioural assay. Within experiment 2, the type of noise treatment had a significant effect on the proportion of successful foraging trials (repeated measures ANOVA, *F*_3,21_ = 17.45, *p* < 0.0001; figure 3*a*) and, again stronger, on the bats' search time (*F*_3,21_ = 82.53, *p* < 0.0001; figure 3*b*). Post hoc tests revealed that the bats' performance did not differ between the digitally generated 15 m stimulus and the ‘continuous’ version of the recorded traffic noise (figure 3). This confirms that the digitally generated noise stimuli we had used in experiment 1 realistically mimicked traffic noise. By contrast, the search time of the bats was more strongly increased under the digitally generated 15 m noise than under the ‘transient’ version of the recorded traffic noise (figure 3*b*). This indicates that the bats were at least to some degree released from acoustic masking between passing vehicles, where the noise intensity and especially the high-frequency content dropped (figure 1*d*).

## 4. Discussion

We assume that search time, as measured in our experiments, is directly related to foraging efficiency in the wild. A long search time indicated that the bats had to fly close to the respective platform to still detect and localize the faint prey rustling sound in noise, i.e. they experienced a reduced detection distance under traffic noise. Our measurements are likely to be conservative estimates, i.e. they probably overestimate the true detection distance. This is because the bats circled above the experimental foraging area in the laboratory and thus passed close to every platform repeatedly, while in the field, mouse-eared bats typically forage in linear flight about 1 m above ground and pass every potential prey only once. If the fivefold increase in search time between control condition and just next to the highway (7.5 m) thus is assumed to indicate a fivefold reduction in detection distance, we would predict a roughly 25-fold decrease in the surveyed ground area for a wild bat. This effect levels off with distance from the highway. Extrapolation of our results suggests traffic noise effects on the bats' prey detection ability up to about 60 m from the highway, which is not a very large distance. However, considering the hundreds of thousands of kilometres of motorways on our planet [1,2], a strip of 50 to 60 m left and right of the tarmac adds up to considerable areas that will be degraded in their suitability as foraging habitats for acoustic predators such as bats and owls. It is important to note that our treatment in experiment 1 mimicked the acoustic situation when a vehicle is passing a foraging bat. The extrapolation of our results to estimating degradation of foraging habitat quality alongside highways owing to noise pollution thus requires taking traffic density into account.

We hypothesize that the mechanistic reason for the deterioration of the bats' foraging performance in noise was acoustic masking [4]; i.e. the loud traffic noise interfering with the perception of the faint prey rustling sound as a separate stimulus. An alternative, but mutually non-exclusive explanation is that the bats' attention was distracted from the prey sounds by the noise background. An animal's attention, i.e. the neuronal representations activated at any given time, is limited, and this can result in important fitness consequences with respect to foraging or vigilance against predators [27]. As an example, Chan *et al*. [14] showed that boat motor noise may distract the finite attention of hermit crabs from approaching predator dummies. Also bats appear to experience some difficulty in processing more than one stream of information at a time [28]. However, in our experiments, we did not observe any sign for a shift of the bats' attention from search for prey cues to the noise; at least not on a behavioural level. During noise treatments, they did not approach or inspect the speakers that were located at the sides of the foraging arena. Rather, they showed the same type of search flight above the feeding platforms as during the silence treatment. The better performance of the bats under transient as compared with continuous traffic noise also indicates that masking and not distraction might have been the main factor. This is because it is unlikely that attention would have fully refocused on foraging in the short intervals between car passes, whereas release from masking can happen within milliseconds [29]. While we therefore consider masking to be the predominant mechanistic cause, we cannot exclude that distraction may play some role for explaining our results as well. Regardless, none of these mechanistic explanations would in any way affect our main empirical result and its ecological implications: bat prey detection performance deteriorates under traffic noise, which might alter predator–prey dynamics and affect ecosystem processes.

As mouse-eared bat echolocation calls are dominated by frequencies between 25–120 kHz [30], there is little overlap with traffic noise and hence hardly any potential for acoustic masking of echoes. Indeed, we had no indication that the bats' orientation by echolocation was impaired. They navigated the flight room and showed well-controlled approach flights to the landing platforms under all noise treatments. Yet, it is known that bats can adapt time–frequency structure and amplitude of their echolocation pulses to interfering noise if it overlaps frequencies crucial for echo imaging [31–33].

Despite the clear noise effect on foraging efficiency, it is astonishing to note that the bats performed way above chance level even at 7.5 m from the highway; they still detected and localized the rustling sound under intense traffic noise in about 50 per cent of the trials. Bats and other acoustic predators are to some degree evolutionarily adapted to foraging under natural noise such as wind or running water. Traffic noise does thus not constitute a completely new situation [34], but it confronts animals with unusually high noise levels over large areas of land [2]. As one strategy to reduce noise interference, bats probably make use of the directional characteristics of their ears [35] to achieve some spatial separation between the prey sound from the ground and the traffic noise from the side or ahead. Furthermore, bats may benefit from a disparity in the temporal structure of noise and prey rustling sounds [29]. Prey rustling is transient and click-like and the highest frequency components of these clicks exceed the traffic noise band [24,25], which again explains why the bats had to pass very close to the prey in strong noise to still hear these quickly attenuating high frequency components. In exceptional cases, natural noise can be more similar to prey rustling in time and frequency structure and thus even have stronger masking effects than traffic noise. One example is the click-like noise produced by wind-moved reeds (B. M. Siemers & A. Schaub 2008, unpublished data; see [24]).

Our study provides direct experimental evidence that anthropogenic noise can affect the foraging efficiency of acoustic predators such as bats and probably also owls, some nocturnal primates, carnivores and others. Many of those are endangered and protected under national and international law. Through interference with the predators sensory performance or attention, traffic noise can reduce predation pressure [13] and thus alter predator–prey dynamics, which in turn affect other ecological processes and ultimately ecosystem stability. We thus argue that noise emissions and other forms of ‘sensory pollution’ [36] need to be considered for the assessment of environmental impact of human activities.
